# Reappraisal of waist circumference cutoff value according to general obesity

**DOI:** 10.1186/s12986-016-0085-y

**Published:** 2016-04-05

**Authors:** Kyung-Soo Kim, Hyun-Ju Oh, Young Ju Choi, Byung Wook Huh, Soo-Kyung Kim, Seok Won Park, Eun Jig Lee, Yong-Wook Cho, Kap-Bum Huh

**Affiliations:** Department of Internal Medicine, CHA Bundang Medical Center, CHA University, Seongnam, Republic of Korea; Department of Internal Medicine, Bundang Jesaeng General Hospital, Seongnam, Republic of Korea; Huh’s Diabetes Center and the 21th Century Diabetes and Vascular Research Institute, Seoul, Republic of Korea; Department of Internal Medicine, Yonsei University College of Medicine, Seoul, Republic of Korea

**Keywords:** Waist circumference, Obesity, Abdominal obesity

## Abstract

**Background:**

Current criterion of waist circumference (WC) for abdominal obesity is not enough to demonstrate characteristics of obese and non-obese populations defined by BMI. The aim of this study was to redefine the cutoff values of WC according to general obesity (BMI ≥ 25 kg/m^2^).

**Methods:**

The receiver operating characteristic curve analysis was performed to determine cutoff values of WC for predicting atherosclerosis according to BMI in 1,063 non-diabetic subjects. To validate this new criterion, diabetic patients (*n* = 3,690) were divided into three groups based on the current (WC of 90/80 cm for men/women) and new cutoff values of WC: 1) group with WC below the lowest value of two criteria; 2) intermediate group defined as having a WC between them; and 3) group with WC more than the highest value of them.

**Results:**

The new cutoff values of WC for predicting atherosclerosis in non-diabetic subjects were 84/76 cm for non-obese men/women, and 93/87 cm for obese men/women, respectively. Of non-obese diabetic patients, the intermediate group (WC 84 ~ 90/76 ~ 80 cm for men/women) was more insulin resistant and showed elevated odds ratio (OR) for having 2 or more metabolic risk factors compared to group with WC below 84/76 cm for men/women [OR 2.48 (95 % CI 1.89–3.25) in men, 2.01 (95% CI 1.45–2.78) in women]. In contrast, among obese diabetic patients, insulin resistance and the likelihood of having 2 or more metabolic risk factors were not different from the intermediate group (WC 90 ~ 93/80 ~ 87 cm for men/women) and group with WC below 90/80 cm for men/women.

**Conclusions:**

The current universal cutoff values of WC may under- or over-estimate the metabolic risks of intermediate groups. Therefore, the WC criteria for abdominal obesity should be applied differently depending on the BMI.

**Electronic supplementary material:**

The online version of this article (doi:10.1186/s12986-016-0085-y) contains supplementary material, which is available to authorized users.

## Background

Obesity is known as a potent risk factor for metabolic disorder and cardiovascular disease [1]. The relationship between obesity and cardiovascular disease depends not only on the amount of body fat but also on its distribution [2, 3]. Recently, increasing evidence has shown that abdominal obesity is critical risk factor for the development of insulin resistance, metabolic syndrome, type 2 diabetes, and cardiovascular disease [4–7]. Abdominal obesity is defined according to ethnically specific values of waist circumference (WC). The cutoff value for abdominal obesity in the Korean population was defined as ≥ 90 cm for men and ≥ 80 cm for women according to Asian-Pacific guideline [8].

However, without consideration for general obesity, it is not appropriate that we use same WC cutoff value for abdominal obesity. For instance, individuals who have been described as “metabolically healthy obese”, the obese phenotype may exist in the absence of metabolic abnormalities such as dyslipidemia, insulin resistance, hypertension and an unfavorable inflammatory profile [9–11]. Similarly not all non-obese individuals present with a healthy metabolic profile [12]. Such discrepancies between obesity and metabolic abnormalities have been explained by several factors, including fat distribution [13]. It is necessary to separately define the precise criteria for abdominal obesity by WC according to the presence or absence of general obesity. However, to the best of our knowledge, no previous studies have attempted to redefine the cutoff value of WC for abdominal obesity in each obese and non-obese individual. The aim of this study was to redefine the cutoff value of WC for abdominal obesity according to general obesity.

## Methods

### Subjects

In the present study, 1,063 non-diabetic subjects were enrolled who had undergone a medical check-up at the Korea Association of Health Promotion Center in Seoul, Korea. In addition, 3,690 consecutive patients with type 2 diabetes who visited diabetes clinic at the Huh’s Diabetes Center, Seoul, Korea were also enrolled. All participants were aged 18 years or above and individuals with acute disease, known liver or kidney disease, or a history of cancer were excluded. Anthropometric assessments were performed, blood pressure was measured, and laboratory tests and carotid ultrasonography were performed in all study participants. To evaluate insulin resistance in diabetic patients, a short insulin tolerance test (SITT) was performed and assessed by the rate constant for plasma glucose disappearance (*Kitt*). Medical histories were collected through personal interview. All participants signed consent forms and the Institutional Review Board of Severance Hospital at Yonsei University College of Medicine approved this study.

### Clinical and laboratory measurements

Height and weight were measured in all subjects while wearing light clothing and no shoes. WC was measured at the midpoint between the inferior border of the subcostal margin and iliac crest in the mid-axillary line after normal expiration with the subject standing. Blood pressure was measured using a mercury sphygmomanometer in a sitting position after the participants had remained seated for 10 min. Venous blood samples were obtained after an overnight fast of at least 8 h and fasting plasma glucose, total cholesterol, triglyceride, and high-density lipoprotein (HDL)-cholesterol were measured. Hemoglobin A_1c_ (HbA_1c_) was analyzed using high performance liquid chromatography (Variant II, Bio-Rad, CA, U.S.A.) in subjects with type 2 diabetes.

### Assessment of mean carotid artery intima-media thickness (C-IMT)

The common carotid arteries were scanned bilaterally using a high-resolution real-time B-mode ultrasonography (Toshiba SSA-270A, Japan in non-diabetic subjects; LOGIQ 7, GE, Milwaukee, WI, USA, in diabetic subjects) with a 10-MHz linear transducer. Scanning was performed at the mid- and distal-common carotid artery by a lateral longitudinal projection. The C-IMT was measured at three points on the far wall of the mid- and distal-common carotid artery, 1 cm proximal to the dilatation of the carotid bulb, and the mean value of six measurements from the right and left common carotid arteries were used. C-IMT was defined as the distance between the lumen-intima interface and the media-adventitia interface. A plaque was defined as a localized protrusion into the vessel lumen with thickening of the vessel wall of > 50 % compared to the adjacent C-IMT.

### Measurement of insulin resistance

The SITT was carried out at 8.00 a.m., after an overnight fast. With the subject at rest, 0.1 U per kg of body weight of a 100 times diluted short-acting human insulin (Humulin-R, Eli Lilly, IN, U.S.A.) was administered via the vein, and a blood sample was obtained from the opposite vein at 0, 3, 6, 9, 12, and 15 min. Plasma glucose concentrations were determined immediately after sampling using a Beckman glucose analyzer II (Beckman Ins., Fullertone, CA, USA), and then *Kitt* was calculated from the slope of the fall in log transformed plasma glucose between 3 and 15 min. Immediately after the test, 100 mL of 20 % dextrose solution was administered intravenously to avoid potential hypoglycemia.

### Definitions of general obesity, atherosclerosis, and metabolic risk factors

General obesity was defined as a BMI ≥ 25 kg/m^2^. Atherosclerosis was defined as history of coronary artery disease or cerebrovascular disease or presence of plaque or thickened C-IMT greater than 1 SD compared with age & sex matched mean value in non-diabetic subjects [14] (Additional file 1: Table S1). We used the criteria for metabolic risk factors proposed by the modified National Cholesterol Education Program Adult Treatment Panel III definition: high serum triglyceride levels (≥150 mg/dL), high blood pressure (systolic, ≥ 130 mmHg; diastolic, ≥ 85 mmHg; or the use of antihypertensive medications), low serum HDL-cholesterol levels (<40 mg/dL for men, < 50 mg/dL for women) [15].

### Statistical analysis

The receiver operating characteristic (ROC) curve analysis was performed to determine cutoff values of WC yielding the maximum sensitivity and specificity for predicting atherosclerosis in non-diabetic subjects. The new cutoff was chosen by maximizing the sums of the Youden’s index where (sensitivity + specificity)—1.

To validate these new criteria, diabetic patients were divided into three groups based on the current (WC of 90/80 cm for men/women) and new cutoff values of WC according to gender and general obesity: 1) group with WC below the lowest value of two criteria; 2) intermediate group defined as having a WC between them; and 3) group with WC more than the highest value of them. Data for continuous variables are presented as the mean ± SD and categorical factors are reported as percentages. Comparisons between the groups were tested using the chi-Square test or one-way ANOVA followed by Tukey’s b *post-hot* test, as appropriate. Odds ratios (OR) for having 2 or more metabolic risk factors in each group were determined using logistic regression analysis, and the group having WC below the lowest value of current and new criteria was used as the reference. ANCOVA was used to adjust for age, diabetes duration, current smoking, and medication usage in multivariate analysis. A *p* value < 0.05 was considered significant. All statistical analyses were performed using IBM SPSS Statistics (version 19.0; IBM Co., Somers, NY, USA).

## Results

Table 1 shows the clinical and biochemical characteristics of the non-diabetic subjects. The mean age was 51.1 years and the mean BMI was 24.5 kg/m^2^. Approximately 21 % of the men and 25 % of the women were found to have atherosclerosis.

**Table 1:** Clinical and biochemical characteristics of the non-diabetic subjects according to sex

	Men	Women	Total
N	565	498	1063
Age (years)	49.9 ± 11.4	52.5 ± 9.9	51.1 ± 10.8
Height (cm)	168.8 ± 5.7	155.4 ± 5.1	162.5 ± 8.6
Weight (kg)	69.4 ± 9.1	59.6 ± 8.5	64.8 ± 10.1
Body mass index (kg/m^2^)	24.3 ± 2.8	24.7 ± 3.2	24.5 ± 3.0
History of coronary artery disease, n (%)	19 (3.4)	28 (5.6)	47 (4.4)
History of cerebrovascular disease, n (%)	14 (2.5)	14 (2.8)	28 (2.6)
Waist circumference (cm)	86.2 ± 7.5	82.1 ± 8.5	84.3 ± 8.2
Systolic blood pressure (mm Hg)	129.9 ± 17.5	131.0 ± 20.0	130.4 ± 18.7
Diastolic blood pressure (mm Hg)	80.4 ± 11.9	79.9 ± 12.7	80.2 ± 12.3
Fasting glucose (mg/dL)	96.6 ± 14.9	93.3 ± 14.7	95.0 ± 14.9
Total cholesterol (mg/dL)	199.0 ± 33.1	206.1 ± 37.2	202.3 ± 35.2
Triglyceride (mg/dL)	188.8 ± 134.8	148.4 ± 146.2	169.8 ± 141.6
HDL-cholesterol (mg/dL)	44.0 ± 9.8	51.2 ± 13.7	47.3 ± 12.3
C-IMT (mm)	0.68 ± 0.17	0.68 ± 0.16	0.68 ± 0.16
Carotid plaque, n (%)	28 (5.0)	26 (5.2)	54 (5.1)
Atherosclerosis, n (%)	120 (21.2)	126 (25.3)	246 (23.1)

Data are expressed as the mean ± standard deviation or number

*HDL* high-density lipoprotein; *C-IMT* mean carotid artery intima-media thickness

The optimal WC measurements as obtained from ROC curves were used for predicting atherosclerosis in non-diabetic subjects were 84 cm in non-obese men, 76 cm in non-obese women, 93 cm in obese men, and 87 cm in obese women, respectively. These new cutoff values displayed the maximal Youden’s index compared with current cutoff values. Sensitivity, specificity, positive and negative predictive values are also presented in Table 2.

**Table 2:** Comparison of waist circumference cutoff values for abdominal obesity to predict atherosclerosis in non-diabetic subjects

		Cutoff (cm)	Sensitivity (%)	Specificity (%)	PPV (%)	NPV (%)	Youden’s index
Non-obese (BMI < 25 kg/m^2^)	Men	84	57.6	66.9	26.8	88.3	24.5
		90	16.9	95.0	41.7	84.5	11.9
	Women	76	75.4	47.2	28.7	87.2	22.6
		80	46.2	68.0	28.8	81.8	14.2
Obese (BMI ≥ 25 kg/m^2^)	Men	90	68.9	37.8	29.2	76.5	6.7
		93	44.3	67.7	33.8	76.6	12.0
	Women	80	98.4	8.5	31.7	92.3	6.9
		87	70.5	53.2	39.4	80.6	23.7

*PPV* positive predictive value; *NPV* negative predictive value

When applying the current and new cutoff values of WC in diabetic patients, the intermediate group was defined as having WC between 84 ~ 90 cm/76 ~ 80 cm for non-obese (BMI < 25 kg/m^2^) men/women, or having WC between 90 ~ 93 cm/80 ~ 87 cm for obese (BMI ≥ 25 kg/m^2^) men/women. In non-obese diabetic patients, the intermediate group had higher blood pressure, higher triglyceride and lower HDL-cholesterol levels than the group with WC of < 84/76 cm for men/women. The intermediate group tended to have similar metabolic profiles as the group with WC of ≥ 90/80 cm for men/women. C-IMT was higher in the intermediate group of non-obese diabetic women than that in the group with WC of < 76 cm. Fasting glucose, HbA_1c_, and the frequency of atherosclerosis did not different among each group (Table 3). In contrast, among obese diabetic patients, metabolic components such as fasting glucose, blood pressure, and lipid profiles were similar in the three groups, except systolic blood pressure in obese men and HbA_1c_ and triglyceride in obese women. C-IMT and the frequency of atherosclerosis did not differ significantly between the intermediate group and the group with WC of < 90/80 cm (Table 3).

**Table 3:** Clinical characteristics of diabetic patients according to sex, waist circumference (WC) and BMI

Men	Non-obese (BMI < 25 kg/m^2^)			Obese (BMI ≥ 25 kg/m^2^)		
	WC < 84 cm	WC 84~90 cm (intermediate)	WC ≥ 90 cm	WC < 90 cm	WC 90~93 cm (intermediate)	WC ≥ 93 cm
N	839	347	56	398	166	309
Age (years)	55.2 ± 10.8	57.5 ± 10.1	60.5 ± 9.1^*, **^	52.9 ± 10.0	53.6 ± 11.1	55.1 ± 12.7
Body mass index (kg/m^2^)	22.1 ± 1.8	23.8 ± 0.9^*^	24.2 ± 0.7^*^	26.2 ± 1.0	27.0 ± 1.2^*^	28.8 ± 2.6^*, **^
Waist circumference (cm)	78.5 ± 4.6	86.9 ± 1.7^*^	92.7 ± 2.0^*, **^	87.1 ± 2.5	92.0 ± 0.8^*^	98.5 ± 5.0^*, **^
Systolic blood pressure (mm Hg)	128.5 ± 17.6	133.3 ± 16.9^*^	139.4 ± 17.7^*, **^	134.3 ± 16.6	135.0 ± 15.1	138.1 ± 15.3^*, **^
Diastolic blood pressure (mm Hg)	83.1 ± 11.0	86.4 ± 10.9^*^	89.7 ± 11.9^*, **^	88.6 ± 11.2	89.0 ± 10.2	89.9 ± 11.6
Fasting glucose (mg/dL)	163.2 ± 67.3	158.7 ± 53.2	145.8 ± 47.0	151.8 ± 49.9	149.2 ± 47.7	152.5 ± 53.3
HbA_1C_ (%)	8.5 ± 2.3	8.3 ± 1.8	7.9 ± 1.1	8.0 ± 1.7	7.9 ± 1.4	8.2 ± 1.9
HbA_1C_ (mmol/mol)	69 ± 25	67 ± 20	63 ± 12	64 ± 19	63 ± 15	66 ± 21
Total cholesterol (mg/dL)	186.5 ± 39.7	189.2 ± 34.8	193.5 ± 44.7	185.9 ± 37.9	185.8 ± 35.8	190.2 ± 36.5
Triglyceride (mg/dL)	117.8 ± 71.8	153.8 ± 83.2^*^	164.2 ± 92.5^*^	154.7 ± 84.6	151.9 ± 69.4	162.3 ± 71.1
HDL-cholesterol (mg/dL)	51.8 ± 14.1	46.4 ± 12.1^*^	45.4 ± 13.2^*^	45.5 ± 10.7	46.0 ± 10.5	44.8 ± 10.8
C-IMT (mm)	0.85 ± 0.19	0.88 ± 0.20	0.91 ± 0.20^*^	0.84 ± 0.18	0.86 ± 0.19	0.89 ± 0.19^*^
Diabetes duration (year)	8.8 ± 8.1	8.6 ± 7.7	8.4 ± 7.2	6.8 ± 6.4	6.8 ± 7.0	6.5 ± 7.2
Atherosclerosis (%)	362 (43.1)	156 (45.0)	25 (44.6)	175 (44.0)	81 (48.8)	156 (50.5)
Anti-hypertensive medication (%)	179 (21.3)	94 (27.1)	22 (39.3)^***^	110 (27.6)	61 (36.7)	120 (38.8)^***^
Statin (%)	59 (7.0)	36 (10.4)	6 (10.7)	41 (10.3)	23 (13.9)	45 (14.6)
Women	Non-obese (BMI < 25 kg/m^2^)			Obese (BMI ≥ 25 kg/m^2^)		
	WC < 76 cm	WC 76~80 cm (intermediate)	WC ≥ 80 cm	WC < 80 cm	WC 80~87 cm (intermediate)	WC ≥ 87 cm
N	506	242	218	69	267	273
Age (years)	57.0 ± 9.5	59.3 ± 9.5^*^	60.2 ± 7.8^*^	57.9 ± 10.8	58.9 ± 9.5	59.8 ± 9.0
Body mass index (kg/m^2^)	21.3 ± 1.9	23.1 ± 1.1^*^	23.8 ± 0.9^*, **^	26.1 ± 1.1	27.0 ± 1.3^*^	29.2 ± 2.8^*, **^
Waist circumference (cm)	71.4 ± 4.3	78.4 ± 1.1^*^	83.9 ± 2.6^*, **^	78.6 ± 1.7	84.4 ± 1.9^*^	92.7 ± 4.6^*, **^
Systolic blood pressure (mm Hg)	131.6 ± 19.3	137.0 ± 17.1^*^	138.6 ± 19.9^*^	138.7 ± 15.6	139.3 ± 17.8	142.8 ± 18.9
Diastolic blood pressure (mm Hg)	81.7 ± 11.4	84.3 ± 10.0^*^	84.8 ± 11.3^*^	85.5 ± 9.3	85.1 ± 10.8	87.9 ± 11.9
Fasting glucose (mg/dL)	154.6 ± 64.0	157.1 ± 58.7	153.5 ± 53.7	143.1 ± 52.8	147.3 ± 51.1	153.0 ± 54.0
HbA_1C_ (%)	8.1 ± 2.1	8.3 ± 1.7	8.4 ± 1.7	7.8 ± 1.8	8.0 ± 1.7	8.3 ± 1.6^*^
HbA_1C_ (mmol/mol)	66 ± 23	67 ± 19	68 ± 19	62 ± 20	64 ± 19	67 ± 18^*^
Total cholesterol (mg/dL)	192.4 ± 39.4	200.4 ± 41.4^*^	202.4 ± 37.1^*^	197.0 ± 34.5	202.4 ± 39.4	203.4 ± 38.5
Triglyceride (mg/dL)	111.0 ± 63.4	146.7 ± 79.3^*^	153.0 ± 78.4^*^	129.2 ± 54.5	147.3 ± 73.6	161.4 ± 73.3^*^
HDL-cholesterol (mg/dL)	58.0 ± 14.7	52.7 ± 13.0^*^	51.8 ± 12.8^*^	50.3 ± 11.9	51.7 ± 11.5	48.9 ± 12.7
C-IMT (mm)	0.80 ± 0.18	0.83 ± 0.16^*^	0.85 ± 0.17^*^	0.82 ± 0.17	0.85 ± 0.19	0.86 ± 0.17
Diabetes duration (year)	8.5 ± 7.2	8.8 ± 7.1	9.4 ± 7.2	6.3 ± 6.6	7.3 ± 6.8	7.8 ± 6.9
Atherosclerosis (%)	214 (42.3)	116 (47.9)	107 (49.1)	34 (49.3)	132 (49.4)	144 (52.7)
Anti-hypertensive medication (%)	130 (25.7)	94 (38.8)	79 (36.2)^***^	23 (33.3)	100 (37.5)	126 (46.2)^***^
Statin (%)	67 (13.2)	40 (16.5)	36 (16.5)	9 (13.0)	35 (13.1)	42 (15.4)

Data are expressed as the mean ± standard deviation or number

*HDL* high-density lipoprotein; *C-IMT* mean carotid artery intima-media thickness

^*^ *P*<0.05 vs. Group with WC of < 84/76 cm for non-obese men/women or group with WC of < 90/80 cm for obese men/women

^**^ *P*<0.05 vs. Intermediate group

^***^ *P*<0.05

**Fig. 1:**
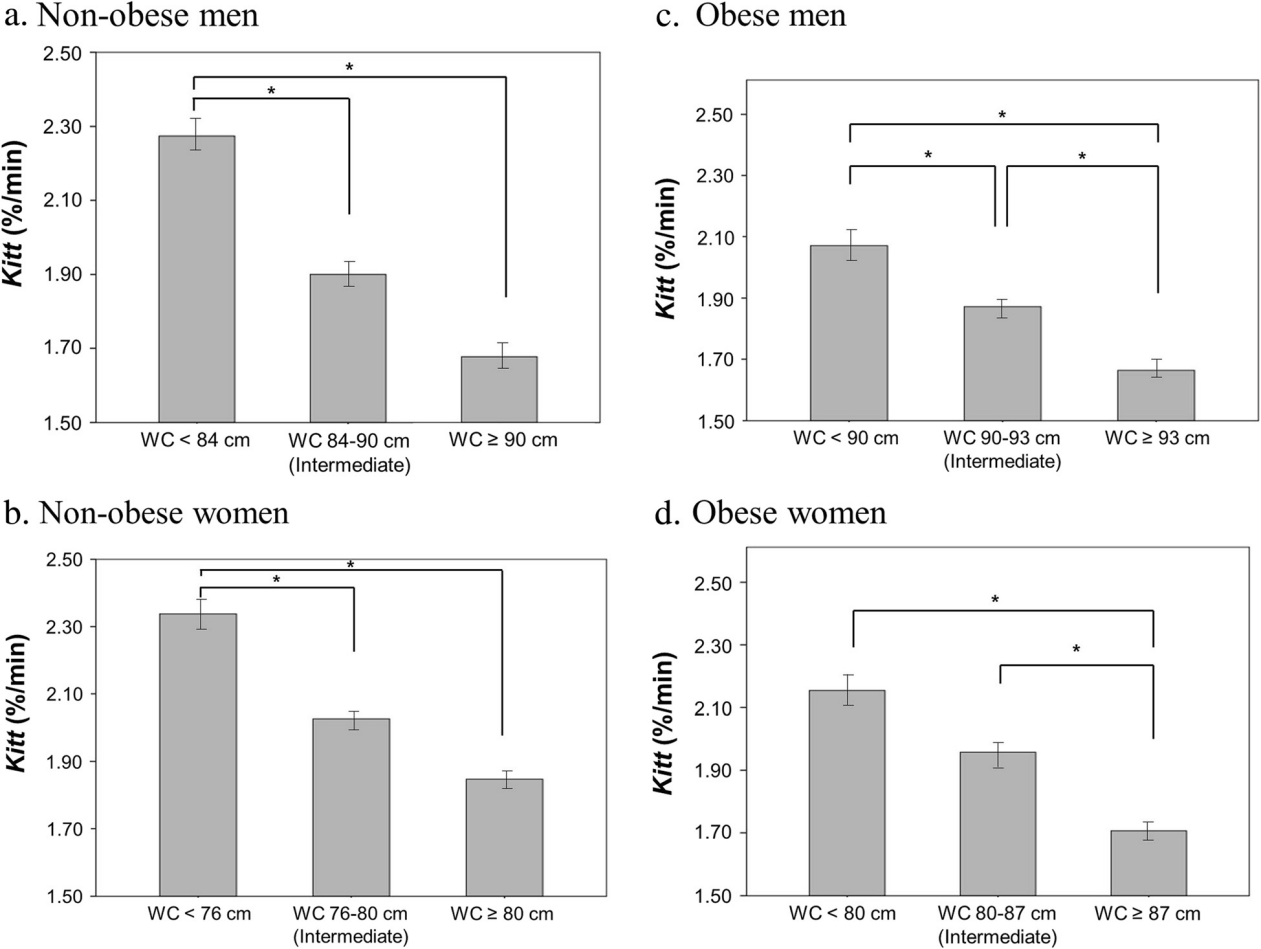
Differences in a *Kitt* (%/min) according to current and new cutoff values of waist circumference (WC) in diabetic patients. **a** in non-obese men; **b** in non-obese women; **c** in obese men; **d** in obese women. In non-obese patients, intermediate group represent lower *Kitt* value compared to group with WC of < 84/76 cm in men/women. In obese patients, intermediate group represent higher *Kitt* value than group with WC of ≥ 93/87 cm in men/women. *, *p* < 0.05

Significant differences were seen in insulin resistance defined by *Kitt* between non-obese diabetic group with WC of < 84/76 cm for men/women and the others, but no difference was found between the intermediate group and the group with WC of ≥ 90/80 cm for men/women (Fig. 1a and b). In obese diabetic patients, the intermediate group had higher *Kitt* values than the group with WC of ≥ 93/87 cm for men/women. *Kitt* did not differ between the intermediate group and the group with WC of < 80 cm for women (Fig. 1c and d).

The ORs for having 2 or more metabolic risk factors according to WC categorized by new and current cutoff values in diabetic patients are shown in Table 4. Of non-obese diabetic patients, the intermediate group were 2.48 times (95 % CI 1.89–3.25) in men and 2.01 times (1.45–2.78) in women, more likely to have 2 or more metabolic risk factors compared to the group with WC below 84/76 cm for men/women, after adjusting for age, diabetes duration, smoking status, and medication usage. In contrast, in obese diabetic patients, ORs were not significantly different between the intermediate group and the group with WC below 90/80 cm for men/women.

**Table 4:** Odds ratios for predicting 2 or more metabolic risk factors in diabetic patients

Men	Non-obese (BMI < 25 kg/m^2^)			Obese (BMI ≥ 25 kg/m^2^)		
	WC < 84 cm	WC 84~90 cm (intermediate)	WC ≥ 90 cm	WC < 90 cm	WC 90~93 cm (intermediate)	WC ≥ 93 cm
Unadjusted OR	1	2.52 (1.94-3.29)	4.65 (2.67-8.10)	1	1.00 (0.70-1.44)	1.44 (1.07-1.94)
Adjusted OR^a^	1	2.48 (1.89-3.25)	4.39 (2.48-7.75)	1	0.96 (0.66-1.39)	1.47 (1.08-2.00)
Women	Non-obese (BMI < 25 kg/m^2^)			Obese (BMI ≥ 25 kg/m^2^)		
	WC < 76 cm	WC 76~80 cm (intermediate)	WC ≥ 80 cm	WC < 80 cm	WC 80~87 cm (intermediate)	WC ≥ 87 cm
Unadjusted OR	1	2.29 (1.67-3.14)	2.91 (2.09-4.03)	1	0.90 (0.53-1.53)	1.72 (1.00-2.94)
Adjusted OR^a^	1	2.01 (1.45-2.78)	2.51 (1.78-3.54)	1	0.91 (0.53-1.57)	1.75 (1.00-3.05)

^a^Adjusted for age, diabetes duration, smoking status, medication usage (insulin, oral anti-diabetic drugs, anti-hypertensive medication, statin)

## Discussion

It has been well known that abdominal obesity and visceral fat, in particular, play an important role in various cardiovascular and metabolic diseases [4–7]. Because BMI does not distinguish the distribution of fat, WC, which is mainly correlated with the distribution of visceral adipose tissue, has been recommended for the specific assessment of abdominal obesity [16–18]. However, current criterion of WC for abdominal obesity is not enough to demonstrate characteristics of obese and non-obese populations defined by BMI. In the present study, we had separately redefined the cutoff value of WC in obese and non-obese population. Our findings may suggest that the current cutoff value of WC underestimated metabolic risks in some non-obese people and overestimated that in some obese people.

In the Asian-Pacific region, the cutoff value for abdominal obesity is defined as ≥ 90 cm for men and ≥ 80 cm for women [8]. The WC cutoffs have been based on available data linking WC with cardiovascular disease and other metabolic syndrome components in different populations [19]. The Korean Society for the Study of Obesity suggested Korean-specific WC cut points of 90 cm for men and 85 cm for women [20]. In addition, WC cutoff values for identifying the presence of insulin resistance and visceral obesity were proposed as 87 cm for men and 81 cm for women in Koreans with type 2 diabetes [21]. However, because abdominal obesity as defined by WC is not enough to capture or explain the metabolic risks for the whole population, other factors such as age and BMI should also be considered. In Japanese-American subjects, a study reported age and gender-specific cut points for abdominal obesity with the International Diabetes Federation criteria. For men, the optimal cut points for WC were 90.0 cm (age < 57 years) and 87.1 cm (age ≥ 57 years). For women, the optimal cut points for WC were 80.8 cm (age < 56 years) and 89.0 cm (age ≥ 56 years) [22]. These results indicated that WC cutoff values were different according to age. Another study on abdominal obesity found different sets of WC values associated with increased risk for cardiovascular disease at the designated BMI values: 90 cm for men and 83 cm for women at 25 kg/m^2^ BMI and 100 cm for men and 93 cm for women at 30 kg/m^2^ BMI [23]. But, to the best of our knowledge, no previous studies have explored WC cutoff values according to general obesity.

Accumulating evidence suggests that not all obese subjects are at increased cardiometabolic risk and not all non-obese are at lower risk. The term, metabolically healthy obesity, has been used to describe an obese phenotype that does not have the burden of any metabolic disorder [9–13]. Conversely, metabolically unhealthy non-obese subjects have been defined as normal BMI and having various metabolic risk factors [12, 13]. Insulin resistance, blood pressure, fasting glucose, lipid profiles, and inflammatory markers are included to define metabolic health but there is no standard definition to discriminate metabolically healthy individuals from those that are metabolically unhealthy [10, 24–26]. In the present study, metabolic profiles such as triglyceride and HDL-cholesterol concentrations and *Kitt* in the intermediate group (WC of 84 ~ 90 cm in men and 76 ~ 80 cm in women) are similar to those in the group with WC of ≥ 90/80 cm for men/women among non-obese diabetic patients. This intermediate group may represent the characteristics of the metabolically unhealthy non-obese population [13, 27]. On the other hand, in the obese diabetic patients, the intermediate group (WC of 90 ~ 93 cm in men and 80 ~ 87 cm in women) had similar metabolic profiles to the group with WC of < 90/80 cm for men/women. This finding is compatible with previously published data on metabolically healthy obese participants [12, 28]. Therefore, in order to distinguish these groups more accurately, it is necessary to redefine WC cutoff values for abdominal obesity based on their general obesity (as defined from BMI).

Similar to the intermediate group in non-obese diabetic patients, metabolically unhealthy non-obese subjects were more commonly found in the Asian population than in Western population [29]. The studies have shown that Asians have a higher level of visceral fat within the same BMI values compared to Caucasians, so the increased risk for metabolic syndrome and cardiovascular disease is present [30, 31]. In a Finnish type 2 diabetes survey, metabolically unhealthy non-obese individuals had higher 2-h postload glucose levels (*p* = 0.003), higher non-alcoholic fatty liver disease scores (*p* < 0.001), and higher cardiovascular disease risk scores (Framingham, *p* < 0.001; SCORE, *p* = 0.002) than metabolically healthy obese individuals [32]. Therefore, it might be necessary to lower WC cutoff values in individuals with normal BMI in order to appropriately manage individuals categorized into intermediate group.

Although there was no universally accepted definition of metabolically healthy obese, many studies have found that individuals with the metabolically healthy obese phenotype are not at increased risk for diabetes, cardiovascular disease, and all-cause mortality [11, 12, 33]. Conversely, metabolically healthy obesity was found to be associated with cardiovascular and all-cause mortality in some studies [25, 34, 35]. Until now, many clinicians have the perception that metabolically healthy obesity is an early stage of the metabolic risk groups. The intermediate group in obese diabetic patients might have been categorized by metabolically healthy obese in aforementioned studies. Therefore close attention should be paid to these patients and redefinition of the WC cutoff values to higher measurements than the current cutoff values to help demonstrate absolutely higher metabolic risk in the obese population should be considered.

One of the strengths in the present study was that we used atherosclerosis instead of metabolic syndrome components to define WC cutoff value for abdominal obesity. Although many studies has used metabolic syndrome components (except WC criteria) to define WC cutoff value, we chose atherosclerosis defined by C-IMT or history of coronary artery disease or cerebrovascular disease because it is a more accurate and precise marker for cardiovascular diseases. In addition, when we verified new WC cutoff values in diabetic patients, we compared insulin resistance calculated by SITT among three groups. SITT is a more accurate method for the evaluation of in vivo insulin sensitivity compared to the homeostasis model assessment of insulin resistance in humans. On the other hand, because this cross-sectional study included only the Korean population, the results cannot be applied directly to other ethnic populations. Our results might be different in other ethnic populations because the Korean population is not obese than Western population. Ultrasonography is highly operator-dependent. In the present study, however, two other sonographers performed the carotid doppler separately in the non-diabetic and the diabetic population, and the intra-observer variability was not checked. The present study investigated differences in insulin resistance and the likelihood of having poor metabolic profiles among groups, but not differences in specific atherosclerotic markers. Moreover, cardiovascular event and all-cause mortality was not fully assessed. Future prospective studies on the risk of suggested WC cutoff values and the incidence of cardiovascular morbidity and mortality are needed.

## Conclusions

In conclusion, the criteria for abdominal obesity defined by current cutoff values of WC may underestimate the metabolic risks in some non-obese people and overestimate that in some obese people. The optimal cutoff of WC may be 84/76 cm in non-obese men/women and 93/87 cm in obese men/women, respectively. The application of these new cutoff values of WC according to BMI may be useful to identify the subjects with poor metabolic profile disproportionate to their underlying BMI and to prevent misclassification some metabolically healthy obese subjects were mistakenly classified as being at high risk. Therefore, we propose that the criteria of WC for abdominal obesity should be applied differently depending on the BMI.

## Additional file

Additional file 1: Table S1. Mean carotid artery intima-media thickness (C-IMT) according to age in non-diabetic subjects in Korea. (DOCX 26 kb)

## Abbreviations

C-IMT: carotid artery intima-media thickness
HbA_1c_: hemoglobin A_1c_
HDL: high-density lipoprotein
OR: odds ratios
ROC: receiver operating characteristic
SITT: short insulin tolerance test
WC: waist circumference
