# Supplementary material for: Reappraisal of waist circumference cutoff value according to general obesity
Source: Nutr Metab (Lond). 2016 Apr 5;13:26. doi: 10.1186/s12986-016-0085-y (PMC4820860; doi:10.1186/s12986-016-0085-y)
Supplement: Additional file 1: Table S1. — Mean carotid artery intima-media thickness (C-IMT) according to age in non-diabetic subjects in Korea. (DOCX 26 kb) [file 12986_2016_85_MOESM1_ESM.docx]

**Table S1** Mean carotid artery intima-media thickness (C-IMT) according to age in non-diabetic subjects in Korea

|  | C-IMT (mm) | |
| --- | --- | --- |
| Age (years) | Men | Women |
| 30~39 | 0.57 ± 0.09 | 0.57 ± 0.10 |
| 40~49 | 0.61 ± 0.11 | 0.60 ± 0.10 |
| 50~59 | 0.72 ± 0.21 | 0.67 ± 0.12 |
| 60~69 | 0.77 ± 0.15 | 0.71 ± 0.13 |
| 70~79 | 0.88 ± 0.18 | 0.76 ± 0.20 |

Data are expressed as the mean ± standard deviation.
